# Prevalence and Molecular Characterization of Glucose-6-Phosphate Dehydrogenase Deficiency at the China-Myanmar Border

**DOI:** 10.1371/journal.pone.0134593

**Published:** 2015-07-30

**Authors:** Qing Li, Fang Yang, Rong Liu, Lan Luo, Yuling Yang, Lu Zhang, Huaie Liu, Wen Zhang, Zhixiang Fan, Zhaoqing Yang, Liwang Cui, Yongshu He

**Affiliations:** 1 Department of Cell Biology and Medical Genetics, Kunming Medical University, Kunming, Yunnan Province, China; 2 The First Affiliated Hospital, Kunming Medical University, Kunming, Yunnan Province, China; 3 Department of Pathogen Biology and Immunology, Kunming Medical University, Kunming, Yunnan Province, China; 4 Department of Entomology, The Pennsylvania State University, University Park, Pennsylvania, United States of America; Université Pierre et Marie Curie, FRANCE

## Abstract

Glucose-6-phosphate dehydrogenase (G6PD) deficiency is an X-linked hereditary disease that predisposes red blood cells to oxidative damage. G6PD deficiency is particularly prevalent in historically malaria-endemic areas. Use of primaquine for malaria treatment may result in severe hemolysis in G6PD deficient patients. In this study, we systematically evaluated the prevalence of G6PD deficiency in the Kachin (Jingpo) ethnic group along the China-Myanmar border and determined the underlying G6PD genotypes. We surveyed G6PD deficiency in 1770 adult individuals (671 males and 1099 females) of the Kachin ethnicity using a G6PD fluorescent spot test. The overall prevalence of G6PD deficiency in the study population was 29.6% (523/1770), among which 27.9% and 30.6% were males and females, respectively. From these G6PD deficient samples, 198 unrelated individuals (147 females and 51 males) were selected for genotyping at 11 known *G6PD* single nucleotide polymorphisms (SNPs) in Southeast Asia (ten in exons and one in intron 11) using a multiplex SNaPshot assay. Mutations with known association to a deficient phenotype were detected in 43.9% (87/198) of cases, intronic and synonymous mutations were detected alone in 34.8% (69/198) cases and no mutation were found in 21.2% (42/198) cases. Five non-synonymous mutations, Mahidol 487G>A, Kaiping 1388G>A, Canton 1376G>T, Chinese 4 392G>T, and Viangchan 871G>A were detected. Of the 87 cases with known deficient mutations, the Mahidol variant was the most common (89.7%; 78/87), followed by the Kaiping (8.0%; 7/87) and the Viangchan (2.2%; 2/87) variants. The Canton and Chinese 4 variants were found in 1.1% of these 87 cases. Among them, two females carried the Mahidol/Viangchan and Mahidol/Kaiping double mutations, respectively. Interestingly, the silent SNPs 1311C>T and IVS11nt93T>C both occurred in the same 95 subjects with frequencies at 56.4% and 23.5% in tested females and males, respectively (*P*<0.05). It is noteworthy that 24 subjects carrying the Mahidol mutation and two carrying the Kaiping mutation also carried the 1311C>T/IVS11nt93T>C SNPs. Further studies are needed to determine the enzyme levels of the G6PD deficient people and presence of additional G6PD mutations in the study population.

## Introduction

Glucose-6-phosphate dehydrogenase (G6PD) deficiency, or “primaquine sensitivity”, is the most common genetic disorder with an estimated 400 million people affected worldwide [1]. The *G6PD* gene spans 18 kb on the X chromosome (Xq28), containing 13 exons and 12 introns [2]. Being X-linked, G6PD deficiency occurs more frequently in males than in females. In hemizygous males and homozygous females, G6PD deficiency is fully expressed, whereas in female heterozygotes, a mixed population of normal and enzyme-deficient cells can be found, owing to random inactivation of one of the two X chromosomes early in embryonic life (Lyonization) [3]. G6PD enzyme catalyzes the production of nicotinamide adenine dinucleotide phosphate (NADPH) in the pentose phosphate pathway. NAPDH is needed for the regeneration of reduced glutathione, the major antioxidant defense, which is particularly important in red blood cells (RBCs). G6PD gene is extraordinarily polymorphic with more than 400 variants discovered based on biochemical diagnosis [4], among which 186 mutations are associated with G6PD deficiency [5]. Most of these mutations reduce the G6PD enzyme stability and activity [6,7]. As a result, G6PD deficient RBCs are more susceptible to destruction by oxidative stress such as oxidant food (fava beans) and drugs (e.g., primaquine and sulfones). Therefore, the disease is often manifested as acute hemolytic anemia triggered by the intake of these stressors.

G6PD deficiency is often quoted as an example of natural selection, with malaria being considered a major evolutionary force. The geographical distribution of G6PD deficiency variants remarkably overlaps with historically malaria-endemic areas such as Africa, Asia, and Mediterranean region [1]. In addition, certain G6PD deficiency variants such as the African A– form and Southeast Asian Mahidol mutation 487A confer certain degrees of protection against severe falciparum malaria and reduce *Plasmodium vivax* parasite densities, respectively [8–10]. The selective advantage against malaria has enabled the persistence of G6PD deficiency in human populations with many variants reaching high frequencies. Consequently, the prevalence of G6PD deficiency is high in malaria endemic areas, ranging from 5% to 20% in Asia and Africa or even higher in some communities [1]. Furthermore, G6PD variants have apparent geographic patterns [11]. For example, the G6PD A^– 202A^ mutation is the predominant one in Africa, while the Mediterranean variant is most widespread in west Asia. In Southeast Asia, however, there is significant heterogeneity of dominant variants in different areas, which appears to be closely related to the geographic locations and associated with different racial and ethnic groups. For example, the G6PD Mahidol 487G>A mutation is considered the predominant variant in Myanmar and present in 91.3% of G6PD deficient patients in study populations [12]. G6PD Kaiping 1388G>A and Canton 1376G>T are the most common variants in southern China with 4–16% prevalence [13]. Thus, the genetic makeup of the human populations in this region may reflect the past history of exposure to malaria as well as genetic isolations of different ethnic groups.

In the Greater Mekong Subregion (GMS) of Southeast Asia including China’s Yunnan Province, Cambodia, Laos, Myanmar, Thailand and Vietnam, malaria is still a major public health problem, and is particularly serious along the international borders [14,15]. In recent years, many nations within this region have been motivated to attempt to eliminate malaria. However, one of the challenges to achieve this goal is vivax malaria, which throughout history has demonstrated tremendous resilience to control efforts. *P*. *vivax* infection produces a dormant form of the parasite called hypnozoite in the liver, which is responsible for subsequent relapses of the disease. Currently, radical cure of vivax malaria requires the administration of the 8-aminoquinoline drug primaquine. However, a key problem with this drug is the risk of hemolysis in patients with G6PD deficiency after primaquine treatment [16]. Since the prevalence of G6PD deficiency varies considerably among the ethnic groups, recommendations for the use of primaquine should be tailored appropriately. Therefore, deployment of primaquine for radical treatment of vivax malaria requires knowledge of the G6PD status in the patient populations.

Various ethnic groups reside in the remote regions along international borders in the GMS, which have been the target populations for WHO’s malaria control programs in past years. Among these ethnicities, the Kachin (Jingpo) lives along the northern part of the China-Myanmar border. Malaria, especially vivax malaria, remains prevalent in this area [17]. In this area, primaquine has been used for radical cure of vivax malaria and recently for eliminating *Plasmodium falciparum* gametocytes. Despite this, systematic studies of the prevalence and genetics of G6PD deficiency among the Kachin population are still lacking. In this study, we surveyed the prevalence of G6PD deficiency in 1770 subjects of the Kachin ethnicity in the China-Myanmar border area using a fluorescent spot test (FST) and determined the G6PD variants in 198 unrelated G6PD deficient subjects.

## Materials and Methods

### Study site and participants

The study was carried out in a settlement for internally displaced people and five villages near Laiza city in northeast Kachin State of Myanmar along the China-Myanmar border. The population living in these border villages mainly consists of the Kachin (Jingpo) ethnicity. In July 2013, 1770 apparently healthy adults of the Kachin ethnicity (no fever or severe anemia), aged 15–65 years, were recruited to this study during active case surveillance of malaria taken place in the catchment area. Some of the study subjects are related; all were included in the calculation of the prevalence of G6PD deficiency. Written informed consent was obtained from adult participants. For minors 15–18 years old, written assent from each participant and his/her parent or guardian was obtained. Ethical approval was obtained from the Research Ethics Committee of Kunming Medical University.

### Blood sample collection and G6PD test

About 0.1 mL of finger-prick blood was collected from each enrolled individual into a tube with EDTA anticoagulant and stored on ice. G6PD enzymatic activity was measured within 12 h using a FST following the manufacturer’s instruction (Micky Ltd, Guangzhou, China). This kit is commonly used to screen G6PD deficiency in newborns in China and recently it was used in an epidemiological screen for G6PD deficiency in Equatorial Guinea [18]. In brief, blood was spotted on Whatman 903 filter paper, air dried, and immediately used for FST. A 3 mm circle of the blood spot was punched out from each sample into a well of a 96-well plate, where 100 μL of reaction reagent was added and incubated at 37°C for 30 min. Three μL of the supernatant was transferred to a filter paper at 10 and 30 min and dried in a 37°C incubator. The formation of the fluorescent product NADPH catalyzed by G6PD was visualized under a UV light (365 nm). Each test included a G6PD normal and G6PD deficient (<40% of normal activity) control. Samples were considered to have normal G6PD activity if fluorescence was shown within 10 min of incubation. In comparison, samples showing fluorescence during 10–30 min of incubation were considered to have intermediate levels of G6PD, whereas samples showing no fluorescence at all after 30 min were considered as G6PD deficient. The last two categories were pooled and collectively designated as G6PD deficient. It is noteworthy that this FST deviates from the Beutler and Mitchell’s original protocol in several steps [19].

### Genotyping of G6PD variants

Genomic DNA was extracted from blood samples with a genomic DNA extraction kit (Takara Biotechnology, Japan) according to the manufacturer's instructions. A multiplex SNAPshot genotyping method was developed for genotyping 11 common mutations in the *G6PD* gene in the GMS [20]. This is essentially a primer extension assay and has been validated by comparison with direct sequencing results of the mutant alleles. The following 11 alleles were determined: 95A>G (Gaohe), 392G>T (Chinese 4), 487G>A (Mahidol), 592C>T (Coimbra), 871G>A (Viangchan), 1024C>T (Chinese 5), IVS11 nt93T>C, 1311C>T, 1360C>T (Union), 1376G>T (Canton) and 1388G>A (Kaiping). 1311C>T is a silent mutation and does not cause amino acid change, while IVS11nt93T>C is located in intron 11. The selection of these two silent mutations for genotyping is based on their relatively high prevalence in Southeast Asia [21–24]. For PCR amplification of the *G6PD* fragments, a 15 μL reaction consisted of 20 ng of genomic DNA, 200 μM dNTP (Takara), 200 pM each primer, 2 U of Platinum Taq DNA Polymerase (Fermentas, Canada), 2 mM MgCl_2_, and 10 × PCR buffer (Mg^2+^ free). The PCR cycle included an initial denaturation step at 95°C for 3 min, followed by 11 cycles of 94°C for 15 s, 62°C for 15 s decreasing 0.5°C every cycle, and 72°C for 30 s, then 24 cycles of 94°C for 15s, 56°C for 15s, 72°C for 30 s, and a final extension step of 72°C for 3 min. Afterwards, 5 μL of the PCR product was analyzed on a 1.5% agarose gel to check for product size and integrity. For the purification of the PCR product from primer contamination, a 7 μL purification reaction consisted of 3 μL of PCR product, 0.8 units of FastAP (Fermentas), and 0.2 μL of 20 u/μL ExoI (Fermentas). The reaction was incubated at 37°C for 15 min and the enzymes were then inactivated at 80°C for 15 min.

The SNaPshot extension reaction was performed using the SNaPshot mix in a PCR machine with 1 μL of SNaPshot ready reaction mix, 0.1 μL of extension primer for each mutation and 2 μL of purified PCR products in a 6 μL total volume. The primer extension reaction used 30 cycles of 96°C for 10 s, 52°C for 5 s, and 60°C for 30 s. Subsequently, 1 μL of the minisequencing products was mixed with 9.5 μL of Hi-Di formamide and 0.5 μL of GeneScan 120 LIZ Size Standard (Applied Biosystems, USA). After denaturing this mixture at 95°C for 3 min and chilling on ice, the fluorescently labeled products were separated by capillary electrophoresis on an ABI PRISM 3730 DNA Sequencer (Applied Biosystems). The data were analyzed using the GeneScan Analysis Software version 3.7.

### Statistical analysis

Statistical analysis was performed using Graphpad Prism (version 6.0). Fisher’s exact test was used for comparisons of frequencies of G6PD deficiency between male and female participants, and for comparisons of allele frequencies between the two sexes.

## Results

### G6PD deficiency in the Kachin population

To determine the prevalence of G6PD deficiency among the Kachin (Jingpo) ethnicity, finger-prick blood samples were obtained from 1770 local inhabitants (671 males and 1099 females) in Kachin State of northeast Myanmar. Diagnosis using the FST identified 523 (29.6%) individuals as G6PD deficient. There was no significant difference in the prevalence of G6PD deficiency between the genders, with 27.9% (187/671) males and 30.5% (336/1099) females being G6PD deficient, respectively (*P* > 0.05; Table 1).

**Table 1:** Prevalence of G6PD deficiency in a Kachin population at the China-Myanmar border.

Gender	# Participants	G6PD deficient [n (%)]	*P* *
Male	671	187 (27.88%)	0.2377
Female	1099	336 (30.57%)	
Total	1770	523 (29.55%)	

*P value shows the difference in the prevalence of G6PD deficiency between males and females as compared using the Fisher’s exact test (two-tailed).

### Identification of G6PD mutations

To determine the genetic basis of G6PD deficiency in the study population, we selected 198 unrelated G6PD-deficient subjects (51 males and 147 females) for genotyping 11 common mutations occurring in Southeast Asia using a recently developed SNaPshot assay. Four G6PD mutations (Gaohe 95A>G, Coimbra 592C>T, Chinese 5 1024C>T and Union 1360C>T) were not detected in this population. Of the 198 individual genotyped, 156 subjects were found to contain mutations in at least one of the 7 remaining loci, whereas 42 (21.2%) subjects did not harbor mutations at any of the sites genotyped (Table 2). Five non-synonymous mutations associated with known G6PD deficient phenotypes were observed in 43.9% (87/198) of the study population. Of these 87 cases, the Mahidol 487G>A type was the most predominant, occurring in 9.7% (78/87) of the subjects. The Kaiping 1388G>A mutation was found in 8.0% (7/87) individuals. Chinese 4 392G >T, Canton 1376 G>T, and Viangchan 871G>A were relatively rare and were detected in one, one and two females, respectively. In addition, the silent mutation 1311C>T and the intron 11 mutation IVS11nt93T>C co-occurred in 58.0% (95/198) of the tested individuals (Table 2).

**Table 2:** Prevalence [n (%)] of G6PD variants in 198 unrelated G6PD-deficient participants^#^.

Mutations	Amino Acid Substitution	Total (n = 198)	Female (n = 147)		Male (n = 51)	*P* *
			Homozygous	Heterozygous		
**Nonsynonymous mutations**						
Chinese 4 392G>T	G131V	1 (0.5%)	0	1 (0.7%)	0	
Mahidol 487G>A	G163S	76 (38.4%)	5 (3.4%)	52 (35.4%)	19 (37.3%)	0.870
Viangchan 871G>A	V291M	1 (0.5%)	0	1 (0.7%)	0	
Canton 1376G>T	R459L	1 (0.5%)	0	1 (0.7%)	0	
Kaiping 1388G>A	R463H	6 (3.0%)	1 (0.7%)	3 (2.0%)	2 (3.9%)	0.000
Mahidol/Viangchan	G163S/V291M	1 (0.5%)	0	1 (0.7%)	0	
Mahidol/Kaiping	G163S/R463H	1 (0.5%)	0	1 (0.7%)	0	
**Synonymous mutations**						
1311C >T/93 T>C**	Silent mutations	95 (48.0%)	9 (6.1%)	74 (50.3%)	12 (23.5%)	0.000
**Unknown**		42 (21.2%)	24 (16.3%)	18 (35.3%)		

^#^ The mutations Gaohe 95 A >G (H32R), Coimbra 592 C >T (R198C), Chinese 5 1024C >T (L342F), and Union 1360 C>T (R454C) were genotyped and not found in this study population.

*P value shows the differences in the prevalence of major G6PD deficiency variants between males and females compared using the Fisher’s exact test (two-tailed).

** 24 and 2 of these double silent mutations co-occurred in females heterozygous for the Mahidol and Kaiping mutations, respectively.

**Fig 1:**
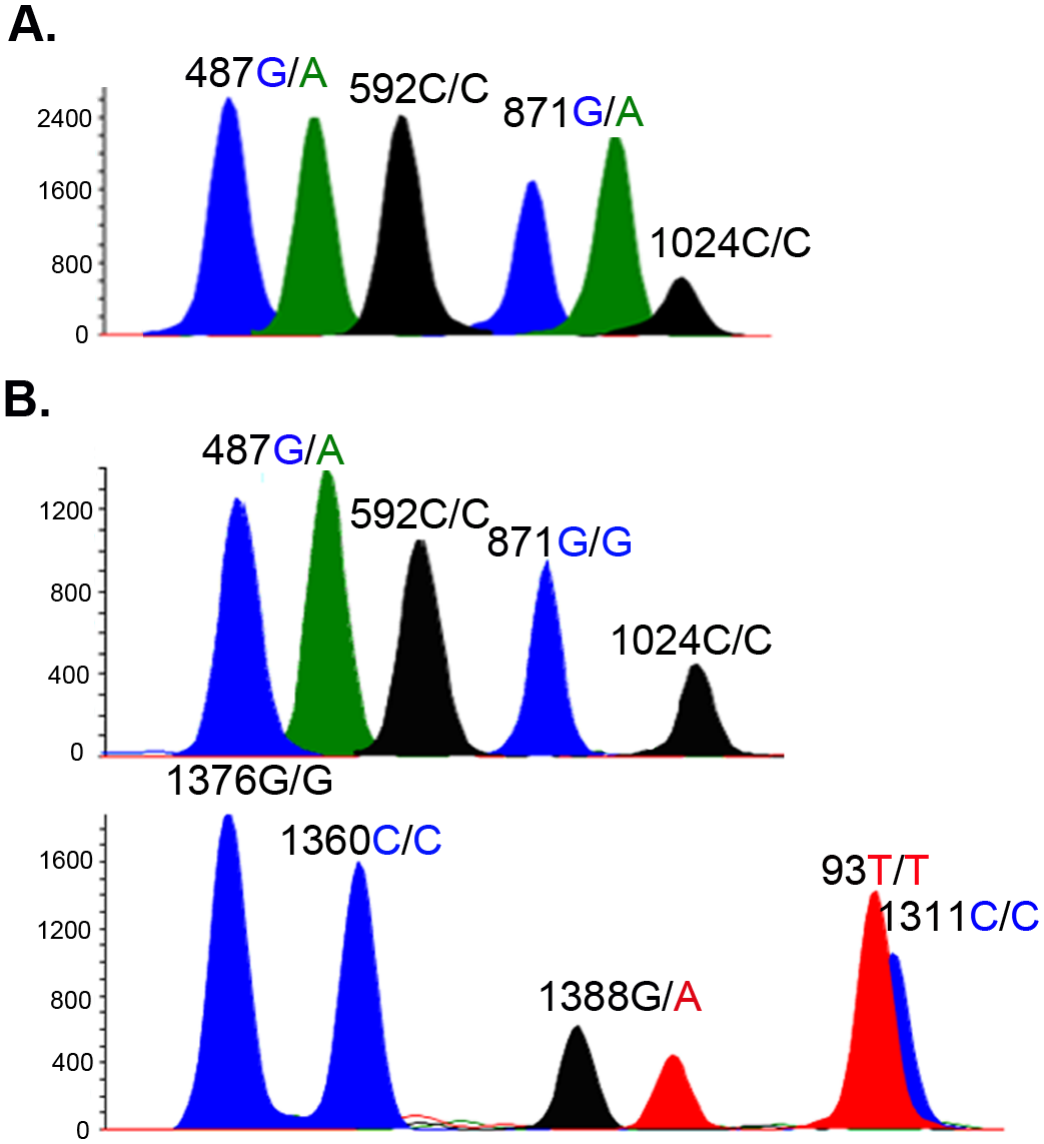
Representative electropherograms of the SNaPshot assay using multiplex extension primers. The homozygous alleles yield only one peak, whereas heterozygous alleles yield double peaks. Female A was heterozygous at both the Mahidol (487G/A) and the Viangchan (871G/A) loci. Female B was heterozygous at the Mahidol (487G/A) and Kaiping (1388G/A) loci.

The female and male populations showed some distinctions in the prevalence and the presence of combined alleles. Whereas all five non-synonymous mutations were detected in the female population, only the two prevalent mutations (Mahidol and Kaiping) were detected in the male population (potentially due to the smaller sample size of the male population). The prevalence of the Mahidol mutation was not significantly different between males and females (*P*>0.05, Fisher’s exact test), being detected in 40.1% (59/147) tested females and 37.3% (19/51) males, respectively. The prevalence of the Kaiping variant was significantly different between tested males and females (*P*<0.001, Fisher’s exact test). In addition, one female was found to contain both Mahidol and Viangchan mutations, while another female harbored both Mahidol and Kaiping mutations (Fig 1, Table 2). Moreover, the two silent mutations were detected alone in 34.8% (69/198) of the cases, whereas 24 (12.1%) and 2 (1.0%) females with the two silent mutations were also found to carry the Mahidol and Kaiping mutations, respectively. In contrast, although the two silent mutations also occurred at a relatively high frequency of 23.5% in the male population, none was found to co-occur with either the Mahidol or the Kaiping mutation (Table 2).

The SNaPshot assay allowed us to distinguish between homozygous and heterozygous status of each allele tested. For the 123 females, we determined whether the mutations were present as heterozygous or homozygous alleles (Table 2). Of the 66 females carrying nonsynonymous G6PD mutations, only 6 (9.1%) were homozygous for the mutations. In particular, the two females that harbored double G6PD mutations were also heterozygous at the two point mutations (Table 2).

## Discussion

The GMS has been a historically malaria hyper-endemic area, and G6PD deficiency has long been recognized as a common inherited hematological disorder in this region [12,25–30]. Here, we evaluated the prevalence of G6PD deficiency along the China-Myanmar border and found a high level (29.6%) of G6PD deficiency in the Kachin (Jingpo) ethnicity. Genotyping at 11 common *G6PD* SNPs present in 198 unrelated G6PD deficient subjects identified 7 of them, among which 5 were non-synonymous mutations. This molecular heterogeneity was expected and is similar to other parts of the GMS [12,13,23,27,29,31,32]. The prevalence of G6PD in the Kachin ethnicity was much higher than those in males of Mon (12%), Burmese (10%), Karen (12.9%), and Burman ethnicities [28,29]. It is also much higher than the prevalence of G6PD deficiency in the Jingpo ethnicity in Yunnan reported earlier [13]. This result could be attributed to differences in sensitivity of the detection kits used, since the FST kit used (Micky Ltd, China) in this study deviates from the original method in several steps [19]. Though this kit has received wide applications in China for screening G6PD deficiency in newborns and recently tested in Africa [18], its sensitivity needs to be evaluated using a quantitative assay.

Numerous studies have shown that the distribution of different G6PD variants is related to geographical regions and ethnic groups [11,27,33]. Factors such as divergent selection forces from different malaria parasite species and geographical separation have caused significant differences in distribution of different G6PD mutations. The G6PD Mahidol 487G>A variant that is associated with reduced *P*. *vivax* parasite density was shown to be under strong positive selection for the last 1500 years [9]. As a result, this mutation has reached significant levels of prevalence in many parts of the GMS. Consistent with findings in the Jingpo ethnicity in Yunnan [13], the Mahidol variant 487G>A was the most common G6PD mutation in the Kachin ethnicity. In addition, Kaiping 1388G>A, Chinese 4 392G>T, Viangchan 871G>A and Canton 1376G>T also occurred in the study population. Interestingly, the Achang, another ethnic group with geographical proximity to the Jingpo, also showed a predominance of the Mahidol mutation (84.2%), though the most dominant mutations Canton 1376G>T and Kaiping 1388G>A found in southern China were not found in the Achang population [34]. An earlier study detected Mahidol mutation as the predominant G6PD mutation in Myanmar [12], despite the inclusion of multiple ethnicities which may have substantial genetic variabilities [35]. Subsequent studies of the Mon, Karen, and Burman ethnicities further corroborated the finding of Mahidol variant as the most common G6PD mutation [28,29]. Especially, the Mahidol mutation has reached 88–96% prevalence in G6PD deficient subjects at the Thai-Myanmar border area [29,30]. This mutation does not seem to impair the catalytic efficiency of the enzyme but rather affects protein folding and stability [7]. This mutation causes a moderate to mild reduction of G6PD activity to 5–32% of wild-type activity in healthy individuals [36], while most patients with the 487G>A variant are usually asymptomatic (with no severe anemia) [37]. Since the Kachin ethnic group also resides in vivax-malaria endemic areas and many of the participants with the G6PD Mahidol mutation were predicted to have <10% residual enzyme activity based on the complete absence of fluorescence by the FST, the standard 14 day primaquine treatment of vivax malaria needs to be administrated with great caution.

The G6PD silent mutation 1311C>T in exon 11, often existing with other G6PD mutations, has been observed in worldwide populations with allele frequencies varying from 0 to 0.45 [38], while in the Indian subcontinent 1311C is usually linked with G6PD Mediterranean 563C>T [39,40]. As a result, this silent mutation is often used as a polymorphic marker in anthropologic and population genetics studies. For example, it is used as a molecular marker to identify G6PD Mediterranean 563C>T mutation origins [39,41]. Geographical variations of this marker may be influenced by gene flow and more than one origin of this mutation. In China and Southeast Asian populations, 1311C>T combined with the IVS11 nt93T>C mutation in intron 11 was reported and it was associated with different ethnic groups [13,41]. Our study identified that 95 G6PD-deficient individuals contained these double mutations, among which 69 have these two mutations only, while 24 and 2 co-occurred with the Mahidol and Kaiping mutations, respectively. The prevalence of these double mutations in the Kachin population is much higher than those previously reported from Yunnan [25] and Thailand [26], but is lower than those from Cambodian [23] and Malaysian Orang Asli populations [42]. Interestingly, an earlier study showed that the Viangchan mutation is responsible for 97.9% of G6PD deficiency in a Cambodian population, and this allele was all linked with the 1311C>T/IVS11 nt93T>C mutations [23]. It is not known whether these silent mutations have any clinical significance.

In this study, 56.1% (111/198) of G6PD-deficient individuals had no identifiable G6PD mutations associated with the deficient phenotype at the tested sites. While we could not exclude the possibility of false “positive” results due to the detection sensitivity of the FST used, it is possible that other untested G6PD mutations or new mutations are present in the study population. For example, the Mediterranean variant 563C>T that is predominant in west Asia has also been observed in Thailand and at the Thai-Myanmar border, albeit at very low frequencies [26,29]. Therefore, inclusion of other G6PD alleles in the SNaPshot assay will provide further resolution of the G6PD genotypes. Sequencing of the *G6PD* gene is necessary to identify uncharacterized novel mutations. Meanwhile, this study also emphasizes that the high proportion of genetically undetermined G6PD deficient individuals really necessitates enzyme activity-based diagnosis of G6PD deficiency before deciding on safety of primaquine treatment.

Malaria elimination is an imminent goal in the China-Myanmar border area. The relatively lengthy course of primaquine treatment required for radical cure of vivax malaria might be a major safety challenge for G6PD deficient people. Most G6PD-deficient individuals in our survey were apparently healthy (with no sign of severe anemia), and genotyping analysis showed that over 80% of the females were heterozygous for the studied G6PD mutant alleles. As a sex-linked trait, it is expected that hemizygous males and homozygous females carrying the G6PD mutations are at a higher risk of hemolytic crisis than in heterozygous females. Although a treatment regimen of 45 mg weekly dose of primaquine for eight weeks was found to be safe and tolerable for radical cure of A^-^ G6PD-deficient patients [43], the safety of this regimen for other G6PD deficient variants have not been tested. Therefore, quantitative analysis of the G6PD activity is required to determine the safety of primaquine administration for radical cure of vivax malaria in people carrying these G6PD deficient variants. With the recent emergence of artemisinin-resistant *P*. *falciparum* in the GMS and potential spread [44,45], it is important to take measures to slow down and prevent further spread of the resistant parasites. A single dose primaquine is advocated for transmission blocking purpose for *P*. *falciparum* malaria. Whereas prospective studies are still needed to confirm that the currently WHO-recommended single-dose primaquine for blocking falciparum malaria transmission confers a very low risk of hemolytic toxicity in G6PD deficient individuals, currently available data suggest that a single dose of primaquine at 0.25 mg base/kg is safe [46,47]. Given the predominant status of the Mahidol mutation in the Kachin population, the use of a single low-dose primaquine for transmission blocking may also be a feasible practice.

In summary, this study is the first report of a systematic survey of G6PD deficiency and associated mutations in the Kachin (Jingpo) ethnic group in the China-Myanmar border area. The prevalence of G6PD deficiency in the study population almost reached 30%. The major mutation associated with the G6PD deficiency was the Mahidol mutation. As malaria elimination campaign is being unfolded in the GMS, G6PD deficiency needs to be monitored before radical cure of vivax malaria is carried out.
