# Correction: Prevalence and Molecular Characterization of Glucose-6-Phosphate Dehydrogenase Deficiency at the China-Myanmar Border

**DOI:** 10.1371/journal.pone.0138038

**Published:** 2015-09-10

**Authors:** Qing Li, Fang Yang, Rong Liu, Lan Luo, Yuling Yang, Lu Zhang, Huaie Liu, Wen Zhang, Zhixiang Fan, Zhaoqing Yang, Liwang Cui, Yongshu He

There are errors in the Results section of this article. In fifth sentence of the “Identification of G6PD mutations” subsection, 9.7% should be 89.7%. The correct sentence is: Of these 87 cases, the Mahidol 487G>A type was the most predominant, occurring in 89.7% (78/87) of the subjects.

In the eighth sentence of the same subsection, 58.0% should be 48%. The correct sentence is: In addition, the silent mutation 1311C>T and the intron 11 mutation IVS11nt93T>C co-occurred in 48% (95/198) of the tested individuals (Table 2).

In addition, there is an error in Table 2: The value 18(35.3%) should not appear in the “Heterozygous” column but in the “Male (n = 51)” column. Please see the correct Table 2 here.

**Table 2:** Prevalence [n (%)] of G6PD variants in 198 unrelated G6PD-deficient participants^#^.

Mutations	Amino Acid Substitution	Total (n = 198)	Female (n = 147)		Male (n = 51)	*P* *
			Homozygous	Heterozygous		
**Nonsynonymous mutations**						
Chinese 4 392G>T	G131V	1 (0.5%)	0	1 (0.7%)	0	
Mahidol 487G>A	G163S	76 (38.4%)	5 (3.4%)	52 (35.4%)	19 (37.3%)	0.870
Viangchan 871G>A	V291M	1 (0.5%)	0	1 (0.7%)	0	
Canton 1376G>T	R459L	1 (0.5%)	0	1 (0.7%)	0	
Kaiping 1388G>A	R463H	6 (3.0%)	1 (0.7%)	3 (2.0%)	2 (3.9%)	0.000
Mahidol/Viangchan	G163S/V291M	1 (0.5%)	0	1 (0.7%)	0	
Mahidol/Kaiping	G163S/R463H	1 (0.5%)	0	1 (0.7%)	0	
**Synonymous mutations**						
1311C >T/93 T>C**	Silent mutations	95 (48.0%)	9 (6.1%)	74 (50.3%)	12 (23.5%)	0.000
**Unknown**		42 (21.2%)	24 (16.3%)		18 (35.3%)	

# The mutations Gaohe 95 A >G (H32R), Coimbra 592 C >T (R198C), Chinese 5 1024C >T (L342F), and Union 1360 C>T (R454C) were genotyped and not found in this study population.

*P value shows the differences in the prevalence of major G6PD deficiency variants between males and females compared using the Fisher’s exact test (two-tailed).

** 24 and 2 of these double silent mutations co-occurred in females heterozygous for the Mahidol and Kaiping mutations, respectively.
